# Spatiotemporal patterns of macrophage migration inhibitory factor (Mif) expression in the mouse placenta

**DOI:** 10.1186/1477-7827-8-95

**Published:** 2010-08-04

**Authors:** Miriam R Faria, Mara S Hoshida, Eloisa AV Ferro, Francesca Ietta, Luana Paulesu, Estela Bevilacqua

**Affiliations:** 1 Institute of Biomedical Sciences, University of São Paulo, São Paulo, Brazil; 2 Department of Morphology, Federal University of Uberlandia, Minas Gerais, Brazil; 3 Department of Physiology, University of Siena, Siena, Italy

## Abstract

**Background:**

Macrophage migration inhibitory factor (MIF) has special pro-inflammatory roles, affecting the functions of macrophages and lymphocytes and counter-regulating the effects of glucocorticoids on the immune response. The conspicuous expression of MIF during human implantation and early embryonic development also suggests this factor acts in reproductive functions. The overall goal of this study was to evaluate Mif expression by trophoblast and embryo placental cells during mouse pregnancy.

**Methods:**

Mif was immunolocalized at implantation sites on gestation days (gd) 7.5, 10.5, 13.5 and 17.5. Ectoplacental cones and fetal placentas dissected from the maternal tissues were used for Western blotting and qRT-PCR assays on the same gestation days.

**Results:**

During the post-implantation period (gd7.5), trophoblast giant cells showed strong Mif reactivity. In later placentation phases (gds 10.5-17.5), Mif appeared to be concentrated in the junctional zone and trophoblast giant cells. Mif protein expression increased significantly from gd7.5 to 10.5 (p = 0.005) and from gd7.5 to 13.5 (p = 0.03), remaining at high concentration as gestation proceeded. Higher mRNA expression was found on gd10.5 and was significantly different from gd13.5 (p = 0.048) and 17.5 (p = 0.009).

**Conclusions:**

The up-regulation of Mif on gd10.5 coincides with the stage in which the placenta assumes its three-layered organization (giant cells, spongiotrophoblast and labyrinth zones), fetal blood circulation begins and population of uNK cells reaches high proportions at the maternal counter part of the placenta, suggesting that Mif may play a role in either the placentation or in the adaptation of the differentiated placenta to the uterus or still in gestational immunomodulatory responses. Moreover, it reinforces the possibility of specific activities for Mif at the maternal fetal interface.

## Background

Macrophage migration inhibitory factor (MIF) is a widely-expressed pleiotropic cytokine, exhibiting a broad range of roles that include pro-inflammatory activities in innate and acquired immunity, glucocorticoid antagonism [1-5], cell proliferation and survival [6-8], cell migration [9,10], modulation of NK-associated immune responses [11], DNA damage response and proteasomal control of the cell cycle [12]. It is constitutively expressed by a wide variety of cells [2,13] and can be either continuously expressed and secreted or stored intracellularly [2].

MIF has been particularly studied during an inflammatory response. Cytokines such as tumor necrosis factor-alpha (TNF-α) and interferon-gamma (IFN-γ) induce MIF expression by macrophages [13] and up-regulation of Toll-like receptors [14], enabling these cells to respond to microbial infection [13-15] and inducing the expression of a large panel of pro-inflammatory molecules (chiefly TNF-α, IFN-γ, interleukin (IL)-1 beta, IL-2, IL-6, IL-8 [1,13]), nitric oxide [16], cyclooxygenase-2 (COX2) products [17] and several metalloproteinases (MMP) [18,19]. Evidence also suggests that MIF inhibits glucocorticoid action by suppressing mitogen-activated protein kinase phosphatase-1 (MKP-1), which activates the proinflammatory extracellular signal-regulated kinase 1/2 (ERK1/2), c-Jun N-terminal kinase (JNK) and p38 pathways [4,20] and inhibits cytokine production.

Activation of the cell surface CD74 by MIF binding initiates a signal transduction cascade resulting in activation of the ERK-1/2 mitogen-activated protein kinase (MAPK) cascade, prostaglandin E2 (PGE_2_) production and cell proliferation [21,22]. However, CD44 seems to be necessary for CD74 signaling [8,23]. Recent data indicate that MIF induces CD44-dependent serine phosphorylation of the intracytoplasmic domain of CD74 and that CD74 and CD44 are associated with the signaling pathway involving Syk tyrosine kinase and phosphoinositide 3-kinase (PI3K)/Akt, leading to cell survival responses and negative regulation of p53, suppressing apoptosis [6,7]. Thus, the functional role of the MIF-activated, CD74-CD44 complex is to deliver important signals for cell survival [8].

The expression of MIF has been described in various organs of the reproductive system in different species [24-27]. In humans, it has been demonstrated in villous and extravillous trophoblast cells and in the endometrium, particularly the glandular epithelium [28-30]. In mice, Mif was identified in the uterus during the pre-implantation period and throughout the estrous cycle as well as in early embryos [24,25]. Mif is also expressed in the trophoblast and maternal epithelium of species with epitheliochorial placentas, e.g. pig [27]. The presence of MIF in the uterus varies during the phases of the reproductive cycle in humans and mice [24,30].

In human pregnancy, MIF has been detected at the site of implantation in both the maternal decidua and trophoblasts [28,31]. It is noteworthy that *MIF *mRNA and protein levels are higher during the very early gestational stages and decline in the late first trimester. MIF neutralization using antibodies increases the cytolytic activity of uterine natural killer cells, suggesting an immunomodulatory role for this cytokine at the maternal-fetal interface [11]. Using an *in vitro *model of chorionic villous explants, we have also found that MIF protein and mRNA are up-regulated by low oxygen tension, comparable to the values during very early stages of pregnancy [31].

As regulatory molecules are important components of a paracrine/autocrine communication network operating within the fetal-maternal unit, we checked for the expression of Mif in fetal placental components. Importantly, our data indicate changes in Mif expression during gestation, which may be associated with placental differentiation and functionality.

## Methods

### Animals

CD-1 mice aged 3-4 months were housed in the animal care facility at the Institute of Biomedical Sciences in the University of São Paulo. Females were caged overnight with males (1:1) and successful mating was checked daily by the presence of a vaginal plug. The morning when the plug was found was designated the first half-day of gestation (gd). A total of 120 pregnant females were used in these experiments.

All pregnant females were killed by cervical dislocation. All procedures and animal handling were performed in accordance with the guidelines provided by the Brazilian College of Animal Experimentation, and were authorized by the Ethical Committee for Animal Research of the University of São Paulo (n° 063/2007).

### Samples

#### Isolation of ectoplacental cones, decidua and fetal placentas

To evaluate protein and gene expression, the uteri were dissected in sterile PBS on gestation day 7.5, immediately after the cervical dislocation. Using a scalpel, the embryo was separated from the decidua and the ectoplacental cone was dissected from the remaining embryonic tissue.

On gd10.5, 13.5 and 17.5, the uteri were opened longitudinally and the fetal placentas were gently isolated from adjacent mesometrial decidua under a stereomicroscope.

#### Implantation sites and placenta

The uteri of females on gd7.5, 10.5, 13.5 and 17.5 were collected immediately after death. The implantation sites (gd7.5) or complete placentas (gd10.5, 13.5 and 17.5) were removed and manually sliced into thin transverse fragments under sterile PBS. Representative samples were immersed in 4% paraformaldehyde in PBS, pH 7.2, for 24 h, followed by dehydration in ethanol and paraffin embedding in Histosec resin (Merck KGaA, Darmstadt, Germany).

### Immunohistochemistry

Paraffin-embedded implantation sites and placentas were cut into 5-μm sections and incubated for 10 min at room temperature with 8% acetic acid to block endogenous phosphatase. Sections were treated with 3% BSA diluted in 0.02 mol/l Tris-buffered saline (pH 7.4) for 30 min at room temperature to block nonspecific binding sites. The samples were incubated for 12 h at 4°C with rabbit polyclonal anti-MIF antibody (Abcam Inc., Cambridge, MA, USA) at 1:100 dilution. The samples were then rinsed in Tris-buffered saline and incubated with a rabbit ExtrAvidin Alkaline Phosphatase Staining kit (Sigma Chemical Co, St. Louis, MO, USA) according to the manufacturer's protocol. The reaction was developed with Fast red AR/Naphtol AS-MX (Sigma Chemical Co, St Louis, MO, USA) and counterstained with Mayer's hematoxylin. Negative controls were performed by replacing the primary antibody with normal rabbit serum.

The immunostaining for each gestational period and each group was determined at different times, assessed by three different observers to obtain the mean scores on a semi-quantitative ranking system, as follow: no staining (-); weak staining (+/-); moderate staining (+); medium staining (++); intense staining (+++). At least five different areas were examined for each section (three sections per group and gestational period) using a light microscope (Nikon, Inc., Tokio, Japan), at the final magnification of ×200.

### Protein and gene expression

#### Collection of samples

Samples were pools of 100 ectoplacental cones randomly obtained from eight pregnant females at gd7.5, and six placentas obtained from three pregnant females (two placentas pooled per female) for each of the remaining days of gestation (10.5, 13.5 and 17.5) per group. A total of three groups were performed for each gestational period. The samples were analyzed in duplicate for qRT-PCR and in triplicate for RT-PCR and Western blotting.

#### Western blotting

The samples were collected on ice-cold RIPA buffer (1% NP-40, 0.25% Na-deoxycholate, 150 mM NaCl, 1 mM EDTA, 1 mM PMSF, 1 mM Na_3_VO_4_, 50 mM Tris-HCl, pH 7.4) supplemented with complete protease inhibitor cocktail (Sigma Chemical Co, St Louis, MO, USA). Thirty μg of total protein were subjected to gel electrophoresis using 15% polyacrylamide gels under denaturing conditions (SDS-PAGE). The separated proteins were electrotransferred to nitrocellulose membranes (Hybond-ECL, GE Healthcare, Buckinghamshire, UK). The blotted membranes were incubated in blocking solution (5% non-fat dry milk in 0.02 M TBS) for 1 h at room temperature and incubated overnight with primary anti-mouse MIF polyclonal antibody (Abcam Inc., Cambridge, MA, USA) at 1:2000 dilution. The membranes were then exposed to horseradish peroxidase-conjugated goat anti-rabbit secondary antibody (KPL Inc., Gaithersburg, Maryland, USA) at 1:2500 dilution. Chemiluminescence was detected using an ECL Chemiluminescent Substrate kit as per the manufacturer's instructions (GE Healthcare, Buckinghamshire, UK). Equal loading of the proteins was confirmed by staining the blots with a 10% Ponceau S solution (Sigma Chemical Co., St Louis, MO, USA). Protein expression levels were determined by densitometry using Scion Image Software (Scion Corp., Frederick, MD, USA).

### RT-PCR and quantitative real-time PCR

#### RNA extraction

For PCR, the samples were dissected in cold sterile PBS. Total RNA was extracted from the samples using TRIzol reagent (Invitrogen, Carlsbad, CA, USA) according to the manufacturer's protocol. RNA concentrations were determined spectrophotometrically by absorbance at 260 nm and purity was assessed by the 260/280 and 230/260 nm ratios and on a 1% denaturing agarose gel stained with ethidium bromide.

#### Oligonucleotide primers

Primers were designed using PrimerQuest Software (Integrated DNA Technologies, Coralville, IA, USA) with reference to GenBank (Table 1).

**Table 1:** Primers used for RT-PCR and qRT-PCR

Primer	**GenBank Accession no**.	Forward 5'-3'	Reverse 5'-3'	PCR(bp) product	T °C
Mif	NM_001111330	TGCCCAGAACCGCAACTACAGTAA	TCGCTACCGGTGGATAAACACAGA	218	60
Cyclophilin	NM_177832.3	CTTGCTGCAGACATGGTC	GCAATCCTGCTAGACTTG	660	58
β-actin	NM_007393	CTGTGGCATCCACGAAACTA	AGTACTTGCGCTCAGGAGGA	199	60

Sequences of oligonucleotide primers, their annealing temperatures (T) and expected product sizes

#### Semiquantitative RT-PCR

First strand cDNA was synthesized from 1 μg total RNA using an Improm-II Reverse Transcription System (Promega Madison, WI, USA) as recommended by the manufacturer.

PCR was performed in a final volume of 25 μl containing 10× PCR buffer, 1.5 mM MgCl_2_, 100 mM deoxynucleotide triphosphates, 100 mM each primer and 0.5 U *Taq *polymerase (Invitrogen, Carlsbad, CA, USA). In addition, several parallel control reactions were run routinely, including RT-PCR in the absence of reverse transcriptase to confirm the absence of genomic DNA contamination, and reverse transcription without RNA to check for reagent contamination. The PCR conditions were: step 1, 94°C for 1 min; step 2, 30 cycles at the annealing temperature indicated in Table 1; and step 3, 72°C for 1 min, using a Mastercycler ep S (Eppendorf, Hamburg, Germany).

The number of cycles used was selected to allow the samples to be compared linearly. *Cyclophilin *was used as the reference housekeeping gene. Table 1 lists the primer sequences, product size and amplification conditions for each gene studied. Total amplification in each reaction primer set was maintained below saturation level so that the products remained within the exponential range. The PCR products were separated by electrophoresis on 1% agarose gels and stained with ethidium bromide.

#### Real-time PCR

To quantify the cDNA generated by reverse transcription, real-time PCR with SYBR Green I was performed using SYBR Green PCR Master Mix in an Applied Biosystems 7500 Fast Real-Time PCR System (both from Applied Biosystems, Foster City, CA, USA). Real-time PCR was carried out using specific primers for MIF and β-actin (Table 1). For negative controls, we used a complete DNA amplification mix in which the target cDNA template was replaced with water. The 2^ΔΔCT method of analysis was used with the β-actin gene for normalization. All samples were run in duplicate in three independent experiments. Amplifications were performed using the default cycling conditions: enzyme activation at 95°C for 10 min, 40 cycles of denaturation at 95°C for 15 s, and annealing/extension at 60°C for 60 s.

To assess the linearity and efficiency of PCR amplification, standard curves for all transcripts were generated using serial dilutions of cDNA. A melting curve was obtained for the amplification products to ascertain their melting temperatures. GeneAmp software (Applied Biosystems, Foster City, CA, USA) was used to quantify the expression levels (Quantitative PCR).

### Statistical analysis

For mRNA expression, normalized cDNA copy numbers for each transcript at different gestation days were compared by ANOVA. The results were considered statistically significant at *P *< 0.05; *P *< 0.1 was considered indicative of possible trends. Spot densitometry was performed to determine Mif band intensities relative to a conserved 70 kDa band detected by Ponceau-S staining [32]. The mean relative ratios and standard deviations (± S.D.) were plotted using Excel for Windows 2000 (Microsoft). Mean values for each gestation period were compared by ANOVA (values statistically significant at *p *≤ 0.05) using Prism for Windows 95, version 4.00 (GraphPad Software Inc.).

## Results

### Immunohistochemistry

**Figure 1:**
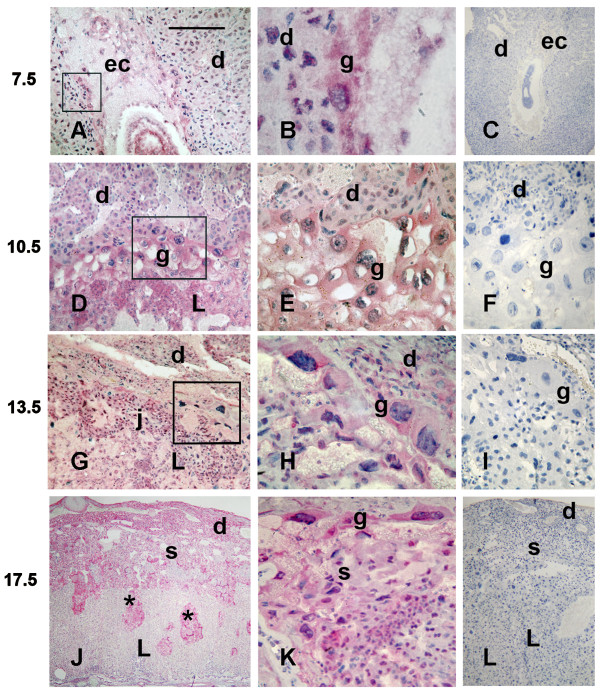
**Immunolocalization of MIF in implantation sites on days 7.5 (A-C), 10.5 (D-F), 13.5 (G-I) and 17.5 (J-L) of gestation**. MIF reactivity (pink to red color) is seen in trophoblast cells and some cells in the mesometrial decidua. Numbers on the left represent gestation days. (ec) Ectoplacental cone; (d) Decidua; (g) Trophoblast giant cells; (j) Junctional zone; (L) Labyrinthine zone; (S and *) Spongiotrophoblast. The boxed areas within low magnifications panels (A, D, G) indicate similar areas of focus in B, E and H. Figures C, F, I and L are negative controls in which the primary antibody was replaced with non-immune serum. Bar in A = 1 mm in C, J and L, 500 μm in A and G, 350 μm in D, I and K, 250 μm in E-F and 150 μm in B and H.

There was Mif immunoreactivity on all gestation days examined (7.5, 10.5, 13.5, and 17.5, Fig. 1). On gestation day 7.5, trophoblast giant cells in the ectoplacental cone (Fig. 1A) and from the mural trophoblast (Fig. 1B) were strongly reactive. Some decidual cells, mainly those located near the embryo, were also immunoreactive. Mif immunostaining was observed in both giant and preplacental cells at gd10.5 (Fig. 1D and 1E), but positive decidual cells were rare. At gd13.5 Mif immunolocalization was restricted to the juncional zone and in some trophoblast giant cells (Fig. 1G and 1H), whereas on gd17.5 the immunolabeling was widespread in these layers (Fig. 1J and 1K). Particularly on gd17.5, spongiotrophoblast characteristically exhibited intrusions into the labyrinth, also immunolabeled for Mif (Fig. 1J).

Immunoreactivity was estimated on a semi-quantitative ranking system: no staining (-); weak staining (+/-); moderate staining (+); medium staining (++); intense staining (+++). The results are summarized in Table 2.

**Table 2:** MIF in placental cell populations

Gestation day	7.5	10.5	13.5	17.5
Trophoblast giant cells	**+**	**+**	**+**	**+**
Spongiotrophoblast		**+**	**+**	**++**
Labyrinth		**+**	**+/-**	**-**

The presence (+) or absence (-) of immunohistochemically detected MIF in placental cell populations at different gestational periods as indicated (++, means many cells immunostained, +/-, only a few reactive cells, - no reactivity).

### Western blotting

**Figure 2:**
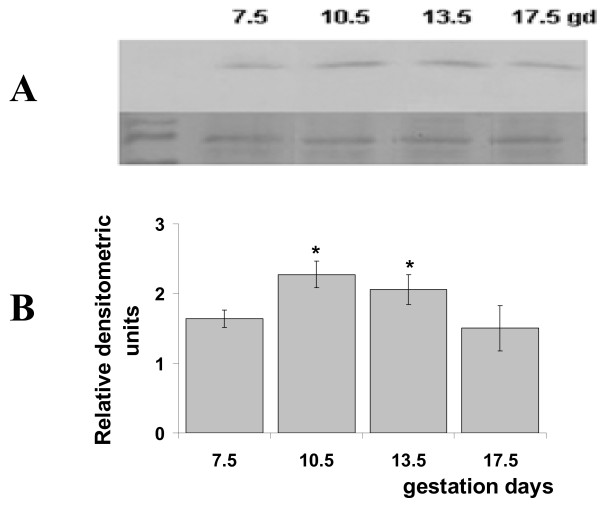
**Western blotting of ectoplacental cone (gd7.5) and placental (gds 10.5, 13.5 and 17.5) homogenates**. The top panel (A) is a representative Western blot showing the detection of MIF protein at different gestational days (numbers on the top). Equal amounts of total cell lysate protein from each sample were separated by SDS-PAGE and immunodetected by Western blotting using anti-mouse MIF antibody. Equivalence of protein loading was confirmed by Ponceau S staining (lower bands). Panel B shows the MIF level by densitometry, presented as mean ± SD of three samples from three separate experiments. *P < 0.05 in relation to the gd7.5.

Mif protein was identified by Western blotting of the homogenates of ectoplacental cones (gd7.5) and placentas (gd10.5, 13.5 and 17.5) (Fig. 2). A specific anti-MIF antibody recognized a single band of approximately 12.5 kDa in all specimens tested (Fig. 2A). The level of Mif expression increased significantly from gd7.5 to 10.5 (*p *= 0.005) and from gd7.5 to 13.5 (*p *= 0.03) (Fig. 2B). As gestation progressed, Mif remained at a high concentration (there were no statistically significant differences among the remaining days of gestation).

### PCR

**Figure 3:**
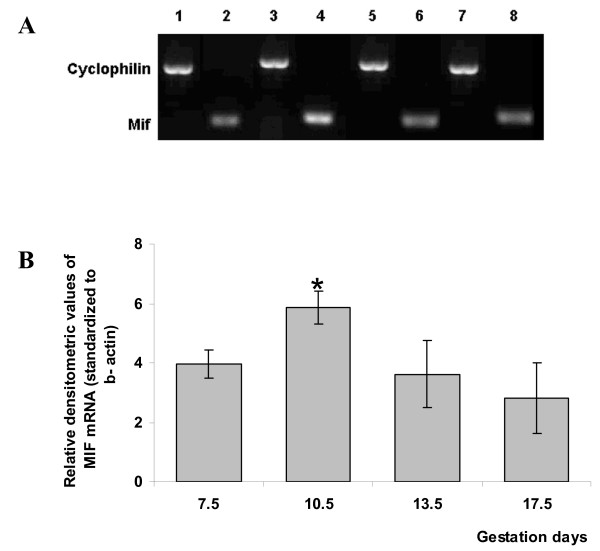
**PCR analysis of *Mif *mRNA in ectoplacental cone and placentas at different gestational periods**. Panel A. Representative agarose gel photograph showing ethidium bromide-stained RT-PCR products of Mif and cyclophilin genes (internal control, cyclophilin). 1 and 2, 7.5 gd; 3 and 4, 10.5 gd; 5 and 6, 13.5 gd; 7 and 8, 17.5 gd. Panel B. *Mif *mRNA expression using RT-qPCR. *Mif *mRNA levels relative to β-actin were measured in all gestational periods indicated using RT-qPCR; the values are given as means ± SEM of three independent experiments. All comparisons were two-tailed. The RT-qPCR results revealed that placentas from gd10.5 show significant up-regulation (*) of *Mif *mRNA levels compared with gd7.5 (*p *= 0.054), 13.5 (*p *= 0.048) and 17.5 (*p *= 0.009).

A band corresponding in size to the *Mif *RT-PCR product was obtained from cDNA in each of the specimens examined (Fig. 3A). *Mif *gene expression was quantified by qRT-PCR (Fig. 3B). The higher mRNA expression was found on gd10.5, and this was significantly different from gds 13.5 (*p *= 0.048) and 17.5 (*p *= 0.009). Indeed, comparison with gd10.5 and gd7.5 showed a possibly significantly increasing trend (p = 0.064).

## Discussion

The post-implantation mouse embryo is completely lined by different types of trophoblast cells with distinct spatial localization and gene expression [33]. The polar trophoblast of the blastocyst gives rise to the ectoplacental cone and chorion trophoblast associated with the development of the fetal counterpart of the placenta [34]. The fusion of the allantois to the chorionic trophoblast originates the labyrinth structure [35] that plays a fundamental role in molecular exchanges between maternal and fetal organisms. The ectoplacental cone originates the trophoblast giant cell layer and junctional zone that comprises both spongiotrophoblast and glycogen cells and is involved with trophoblast proliferation, differentiation and hormone synthesis [36,37]. Glycogen cells also exhibit a migratory pattern into the decidua (from day 12 of gestation) for vessel remodeling and immunomodulatory functions [36,38]. The ectoplacental cone also develops a multitude of giant cells [34] that participate in pivotal processes such as remodeling of the decidua and arterial vessels, immunoregulation and at later stages (after gd 9.5) secretion of regulatory hormones members of the prolactin/growth hormone family of proteins [39]. Moreover, trophoblast giant cells and junctional zone cells establish extensive communication with decidual cells, maternal vascular cells and immune cells.

The present study showed that *Mif *mRNA is detectable in fetal placental components on gestation day 7.5 and its expression increases after gd10.5. Data from gene expression, protein expression and immunolocalization of Mif were consistent through all periods studied. The immunolocalization results also suggested that the main source of Mif at the maternal-placental interface is peripheral giant and junctional zone cells after gd10.5 and trophoblast giant cells from gd7.5 onwards. Coincidentally, day 10 of gestation is also the stage at which the placenta assumes its three-layered organization (giant cells, junctional and labyrinth zones) [38] the fetal blood circulation begins [40], trophoblast cells invade and remodel maternal arterial vessels and uterine killer cells increase the population density at the maternal counterpart of the placenta [41]. The increase in Mif expression and the location at the placental-maternal interface after gd10.5 gathered to the functions previously described for this regulatory factor suggest that Mif may participate in these placentation-associated processes.

One of the most known actions of MIF is its ability in promoting cell proliferation and suppressing apoptosis [6-8]. In fibroblasts, MIF stimulates survival [7]. Coherent with this, MIF mRNA is upregulated in wound healing process [42]. One hypothetical explanation for the high levels of Mif production at maternal-fetal interface may be causally associated with maintenance of decidual cells and, as such, acting as a gestational protective factor.

Several studies provide strong evidence that MIF is a central player in inducing angiogenesis and as a chemoattractant for human vascular endothelial cells [9,10]. Angiogenesis in turn is a fundamental process during chorioallantoic placentation, particularly from embryonic day 10.5, after decline of the vitelline circulation associated with the yolk sac [43]. Thus, it seems reasonable to propose that increased Mif expression on the giant and junctional zone cells, cells in close proximity to the basal decidua and consequently to the maternal vasculature, may participate together other angiogenic factors also produced by these cells with the augment of vessels in the endometrium for placental functioning.

MIF also appears to be an important mediator in the production of extracellular matrix-remodeling factors such as metalloproteinases [18,19] and granzyme B [44]. These enzymes are closely related to migration in trophoblast cells [45-47] and therefore also enable to establish an autocrine correlation with Mif expression and secretion at the maternal counterpart of the placenta. Invasive trophoblast cells are a specialized lineage in rodents [48]. During the second half of gestation these cells exit from the chorioallantoic placenta, invade the mesometrial endometrium to a degree that differs among rodent species and, remodel and colonize the uterine vessels [36,38,49-51].

The overall action of MIF also includes the induction of a large range of pro-inflammatory cytokines (TNF-α, IFN-γ, IL-1β, IL-2, IL-6, IL-8, 2, macrophage inflammatory protein [13]), nitric oxide [16] and COX2 products [17], and counter-regulation of glucocorticoid action on the immune response. Arcuri et al. [28] also argued for a putative immunosuppressor role of MIF, inhibiting uterine natural killer (NK) cell activity in the decidua [11]. The expression of NKG2 D, a NK known activating receptor, is down regulated by MIF decreasing its lytic capacity [52]. In the eye aqueous humor, a site with immune special characteristics as the pregnant uterus, MIF also inhibited NK cell mediate cytolysis in a dose dependent manner by reducing perforin granule exocytoses, in vitro [53]. Interestingly, a coincident profile between the Mif gene/protein expression by fetal placenta components and the variation in the population of uNK cells in the decidua can be observed. Both began to increase by gd7.5, peak about gd10-12 when they decline toward term [current results; [41,54]]. Moreover, Mif immunolocalization showed a consistent pattern in trophoblast giant and spongiotrophoblast cells. Particularly mouse giant cells also secrete a large number of hormones closely related to prolactin (PRL), including the placental hormone prolactin-like protein A (PLP-A) at midgestation [55]. PLP-A specifically interacts with uNK cells, decreases its cytolytic activities [56] and thus, regulates the activity of this class of T lymphocytes at the implantation site. In this context, our findings seem to bolster the use of different programs by trophoblast cells to interact with NK cells and to prepare an adequate immune microenvironment for embryo development.

Immune privileged site can also be induced by progesterone, a best-known mediator at maternal-fetal interface [57]. As MIF and PLP-A, early studies suggest that progesterone can inhibit lymphocyte proliferation and suppress NK cytolytic activity in a dose-dependent manner [58-60]. The progesterone action, however, might also be mediated by MIF. A significant positive correlation has been found between MIF levels and progesterone receptors [61]. In addition, MIF is also a target of sex steroids in some inflammatory models; progesterone increases MIF production in the female rat colon in experimental colitis [62], which may be another reasonable hypothetical triangulation during placental development.

Key cellular MIF functions are mediated through CD74/CD44 receptors and are closely related to the phosphoinositide-3-kinase (PI3K)/Akt signaling pathway [6-8,10]. In this context, the distribution and activation of Mif receptors is now being further investigated in our laboratory. The results may also help to elucidate its paracrine and autocrine actions.

As learned of MIF knockout animals, reproduction is not impaired. However it does not demonstrate that MIF expression can be functionally despicable or unworthy. Genetic redundancy, in which the disruption of one gene is compensated by others leading to no phenotypic effect, is a common finding in different models [63,64] and wouldn't be an isolated example in which different mechanisms would plot to gestation success [63].

In conclusion, our findings provide a global view of Mif expression in trophoblast cells during placental development, highlight correlations among Mif expression, Mif putative functions and important steps of placental development and provide a basis for new approaches to the study of its function(s).

## Competing interests

The authors declare that they have no competing interests.

## Authors' contributions

MRF collected the specimens, performed immunohistochemistry reactions, Western blottings, RT-PCRs and qRT-PCRs, analyzed the data and drafted the manuscript. MSH helped to analyze the data. EAVF helped to design the study and performed the immunohistochemistry reactions. FI helped to design the study and drafted the manuscript. LP helped to design the study and drafted the manuscript. EB participated in the design of the study, helped to analyze the data and was the director of the whole project. All authors read and approved the final manuscript.
